# Pancreatic adenocarcinoma: insights into patterns of recurrence and disease behavior

**DOI:** 10.1186/s12885-018-4679-9

**Published:** 2018-07-28

**Authors:** Ibrahim H. Sahin, Harold Elias, Joanne F. Chou, Marinela Capanu, Eileen M. O’Reilly

**Affiliations:** 1 Emory University School of Medicine, Atlanta, USA; 2 New York University Langone Medical Center, New York, USA; 3 Memorial Sloan Kettering Cancer Center, 300 East 66th Street, Office 1021, New York, NY 10065 USA; 4 David M. Rubenstein Center for Pancreatic Cancer Research, 300 East 66th Street, Office 1021, New York, NY 10065 USA; 5 Weill Cornell Medical College, 300 East 66th Street, Office 1021, New York, NY 10065 USA

**Keywords:** Pancreatic cancer, Lung, Liver, Metastasis, Pre-metastatic niche, Exosomes, Disease behavior, Recurrence pattern, Survival outcomes, Tumor differentiation, Alcohol use, Disease heterogeneity, Chemokine receptors

## Abstract

**Background:**

Pancreatic ductal adenocarcinoma (PDAC) is one of the most aggressive cancers with high metastatic potential. Clinical observations suggest that there is disease heterogeneity among patients with different sites of distant metastases, yielding distinct clinical outcomes. Herein, we investigate the impact of clinical and pathological parameters on recurrence patterns and compare survival outcomes for patients with a first site of recurrence in the liver versus lung from PDAC following original curative surgical resection.

**Methods:**

Using the Memorial Sloan Kettering Cancer Center ICD billing codes and tumor registry database over a 10 years period (January 2004–December 2014), we identified PDAC patients who underwent resection and subsequently presented with either liver or lung recurrence. Time from relapse to death (TRD) was calculated from date of recurrence to date of death. Using the Kaplan-Meier method, TRD was estimated and compared by recurrence site using log-rank test.

**Results:**

The median overall follow-up was 37.3 months among survivors in the entire cohort. Median TRD in this cohort was 10.7 months (95%CI: 8.9–14.6 months). Patients with first site of lung recurrence had a more favorable outcome compared to patients who recurred with liver metastasis as the first site of recurrence (median TRD of 15 versus 9 months respectively, *P* = 0.02). Moderate to poorly or poor differentiation was associated more often with liver than lung recurrence (40% vs 21% respectively, *P* = 0.047). A trend to increased lymph node metastasis in the lung recurrence cohort was observed.

**Conclusion:**

PDAC patients who recur with a first site of lung metastasis have an improved clinical outcome compared to patients with first site of liver recurrence. Our data suggests there may be epidemiologic and pathologic determinants related to patterns of recurrence in PDAC.

## Background

Pancreatic ductal adenocarcinoma (PDAC) has remained a major obstacle for physicians and scientists, due to its distinct underlying molecular behavior and de novo resistance to conventional and targeted therapies. For advanced PDAC, the best survival results are achieved with combination cytotoxic agents such as fluorouracil, irinotecan and oxaliplatin (FOLFIRINOX) [1] or gemcitabine based multi-agent chemotherapeutics [2]. However, the 5-year survival for metastatic disease is 2% which has improved by 1% over the last two decades [3] suggesting limited progress in management of metastatic PDAC. Although gemcitabine-based adjuvant therapy has reduced the recurrence rate of PDAC [4], the 5-year survival of patients who underwent curative resection is estimated to be 27% [3], indicating frequent recurrence in long term follow up ultimately leading to disease-related mortality.

The evidence to date indicates that metastasis is a very sophisticated molecular and cellular process involving distinct signaling pathways that includes crosstalk between cancer cells and the tumor microenvironment [5]. The pre-metastatic niche formation in the host organ may also influence the molecular and clinical behavior of cancer cells [6], indicating that there may be multiple elements that lead to the distinct course of the disease. For example, exosomes, membrane-bound protein and RNA carriers which act as a conduit for cell signals in the tissue microenvironment, may have important functions in the formation of pre-metastatic niche [7]. Tumor-derived exosomes may optimize the distant pre-metastatic microenvironment prior to the occurrence of tumor seeding. A study suggested that mesenchymal-like renal cancer stem cells expressing CD105 release exosomes that facilitate angiogenesis and foster the development of lung metastasis [8]. PDAC-derived exosomes that are received by Kupffer cells have been also shown to create a pre-metastatic niche formation in the liver by recruiting bone marrow-derived macrophages [9]. Collectively, these data indicate that there are inter-site signaling networks between tumor cells and the future host organ to condition the pre-metastatic niche, thereby suggesting that metastasis is an organ-specific, programmed process rather than a random event.

One notable clinical observation is that the heterogeneity of disease behavior of distant recurrent disease following potentially curative surgery, leads to further challenges in optimizing treatment and predicting clinical outcome. In the study reported herein, we focus on patterns of liver and lung recurrence of PDAC and examine the impact of first site of recurrence on disease behavior and post-relapse survival outcomes. We further investigate the potential contributions of epidemiologic and pathologic characteristics on the metastatic pattern of PDAC.

## Methods

### Study population

The institutional cancer registry for Memorial Sloan Kettering Cancer Center and ICD billing codes were queried for PDAC patients (AJCC Stage I/II) who underwent frontline surgical resection and developed either lung or hepatic metastasis as a first-site of recurrence during the 10-year period from January 1, 2004 through December 31, 2014. All patients identified in this query had pathologic confirmation of PDAC diagnosis. Adjudication of metastatic disease was determined by expert clinicians based on a biopsy from metastatic site (where available) and associated clinical and radiologic features. Patients who developed local recurrence, peritoneal carcinomatosis, concurrent multi-organ metastasis or other distant organ dissemination prior to lung and liver metastasis were excluded from the cohort. Patients with the presence of concurrent liver and lung metastasis were also excluded from this analysis. Most of the patients included in the study ultimately developed multisite metastases as their disease progressed. This study was reviewed and approved by the Memorial Sloan Kettering Cancer Center Institutional Review and Privacy Board (IRB) on an annual basis.

### Data collection

Demographic, clinical and pathologic information was obtained from the institutional electronic medical record using the chart review method. The data were collected by trained personnel. Recurrent metastatic disease was defined as the detection of a new distant lesion in the presence of unequivocal clinical and biomarker correlation with or without pathologic confirmation. Microscopic tumor involving the resection margin was considered a positive tumor margin. Tissue diagnosis from the metastatic site along with radiologic and clinical assessment of expert physicians were considered in the decision for adjudication of the site of recurrence of disease. The data, including date of diagnosis, medical and surgical history, pathologic information including tumor differentiation, vascular and perineural invasion, tumor margin status, lymph node status, tumor location and pathologic stage along with social history were obtained by detailed electronic medical record review.

### Statistical analysis

Clinical and pathological characteristics were summarized using frequency and percentages for categorical covariates and mean and standard deviation (SD) for continuous variables and compared between site of recurrence using Fisher’s exact test or Wilcoxon rank -sum test. The time from relapse to death (TRD) was calculated from the date of recurrence to the date of death. Patients alive at the end of the study period were censored at 01/01/2016. TRD was estimated using the Kaplan-Meier method and compared for recurrence sites of liver and lung metastasis using log-rank test. Statistical analyses were performed using SAS Version 9.4 (SAS Institute, INC., Cary, NC, USA). *P*-values were 2-sided and < 0.05 were considered statistically significant.

## Results

**Fig. 1:**
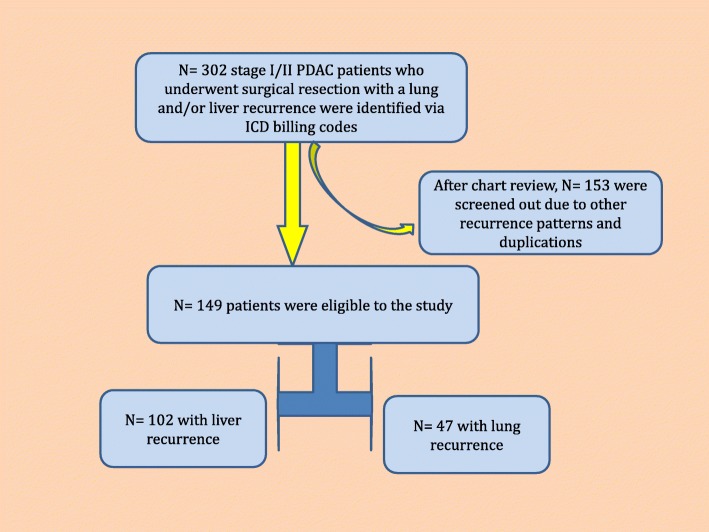
Patient Disposition

A total of *N* = 302 PDAC patients with recurrent/metastatic disease in the liver or lung after initial curative surgery, were identified over the 10-year study period by using ICD and billing codes. After a detailed chart review, *N* = 149 patients were found to have either only liver or lung metastases as the first site of recurrence (Fig. 1). In this cohort of *N* = 149 patients, the majority of the patients were Caucasian and had a normal Body-Mass Index (BMI) at the time of recurrence (Table 1). Most of the patients in the entire cohort had AJCC stage IIB disease at the time of surgical resection and had either a pancreatic head or body tumor. All patients had adenocarcinoma. Eighty percent (*N* = 117) of the patients had lymph node positive disease, 70% (*N* = 103) had vascular invasion, 90.6% (*N* = 135) had perineural invasion. Liver metastasis was the more common site of recurrence (*N* = 102) compared to patients with lung metastasis (*N* = 47). The most common adjuvant therapy administered after initial primary tumor resection was single agent gemcitabine (84%). Other adjuvant therapies utilized in the overall cohort were, fluoropyrimidine-based therapy and fluoropyrimidine-based chemoradiation. The majority of patients received multi-agent regimens at the time of recurrence in various combinations including, FOLFRINOX (5-fluorouracil, oxaliplatin, irinotecan, leucovorin) and gemcitabine-based regimens. In the entire cohort, approximately half of the patients were smokers and had a history of either social or regular alcohol use (Table 2).

**Table 1:** Demographics and treatment history by Recurrence Pattern

	Recurrence Pattern		*p*-value	Entire Cohort
	Liver Metastasis	Lung Metastasis		
Mean Age ±SD	64 ± 11 years	66 ± 10 years	0.43	
Gender			0.99	
Male	55 (54%)	25 (53%)		80 (54%)
Female	47 (46%)	22 (47%)		69 (46%)
Ethnicity			0.80	
Caucasian	86 (84%)	41 (87%)		127 (85%)
Hispanic	5 (5%)	1 (2%)		6 (4%)
African-American	1 (1%)	3 (6%)		4 (3%)
Asian	5 (5%)	1 (2%)		6 (4%)
Others	4 (4%)	–		4 (2%)
N/A	1 (1%)	1 (2%)		2 (2%)
BMI			0.050	
< 18.5	6 (6%)	5 (11%)		11 (8%)
18.5–24.9	40 (39%)	28 (60%)		68 (50%)
25–29.9	35 (34%)	11 (23%)		46 (28%)
30–34.9	15 (15%)	3 (6%)		18 (11%)
≥ 35	6 (6%)	–		6 (3%)
Adjuvant Therapy			0.17	
Gemcitabine-based	77 (75%)	43 (92%)		120 (84%)
Fluoropyrimidine- based	6 (6%)	1 (2%)		7 (4%)
5-FU/Gemcitabine	9 (9%)	2 (4%)		11 (6%)
N/A	10 (10%)	1 (2%)		11 (6%)
Treatment Metastatic Disease				
FOLFIRINOX	18 (18%)	5 (11%)	N/A	23 (15%)
Gemcitabine/Nab-paclitaxel	9 (9%)	1 (2%)		10 (5%)
Other: Single-agent	18 (18%)	11 (23%)		29 (20%)
Other: Multi-agent	31 (30%)	23 (49%)		54 (40%)
N/A (Unknown)	26 (25%)	7 (15%)		33 (20%)

**Table 2:** Clinical and epidemiologic variables by Recurrence Pattern

	Recurrence Pattern		*P* value^*^	Entire Cohort
	Liver	Lung		
Tumor Differentiation^a^				
Well/well to moderate	1 (1%)	1 (2%)	*P* = 0.047	2 (1%)
Moderate	60 (59%)	36 (77%)		96 (65%)
Moderate- poorly/Poorly	40 (40%)	10 (21%)		50 (34%)
Tumor Stage				
Stage I	3 (3%)	–	*P* = 0.194	3 (2%)
Stage IIA	23 (22%)	6 (13%)		29 (19%)
Stage IIB	76 (75%)	41 (87%)		117 (79%)
Lymph Node Status				
Positive	76 (75%)	41 (87%)	*P* = 0.089	117 (79%)
Negative	26 (25%)	6 (13%)		32 (21%)
Vascular Invasion^a^				
Positive	69 (68%)	34 (72%)	*P* = 0.703	103 (70%)
Negative	32 (32%)	13 (28%)		45 (30%)
Perineural Invasion				
Present	90 (88%)	45 (96%)	*P* = 0.226	135 (91%)
Absent	12 (12%)	2 (4%)		14 (9%)
Tumor Margin				
Negative	89 (87%)	36 (77%)	*P* = 0.148	125 (84%)
Positive	13 (13%)	11 (23%)		24 (16%)
Tumor Location				
Head	84 (83%)	38 (81%)	*P* = 0.942	132 (82%)
Body	7 (7%)	4 (8%)		11 (7%)
Tail	11 (10%)	5 (11%)		16 (11%)
Alcohol Use				
None	56 (55%)	15 (32%)	*P* = 0.011	71 (56%)
Social	29 (28%)	25 (53%)		54 (36%)
Daily/heavy	17 (17%)	7 (15%)		24 (16%)
Smoking				
None	55 (54%)	23 (49%)	*P* = 0.60	78 (52%)
Current/former	47 (46%)	24 (51%)		71 (48%)

Note: ^*^p-value was calculated using Fisher’s exact test, ^a^Tumor differentiation and vascular invasion data was missing for one patient in liver group

**Fig. 2:**
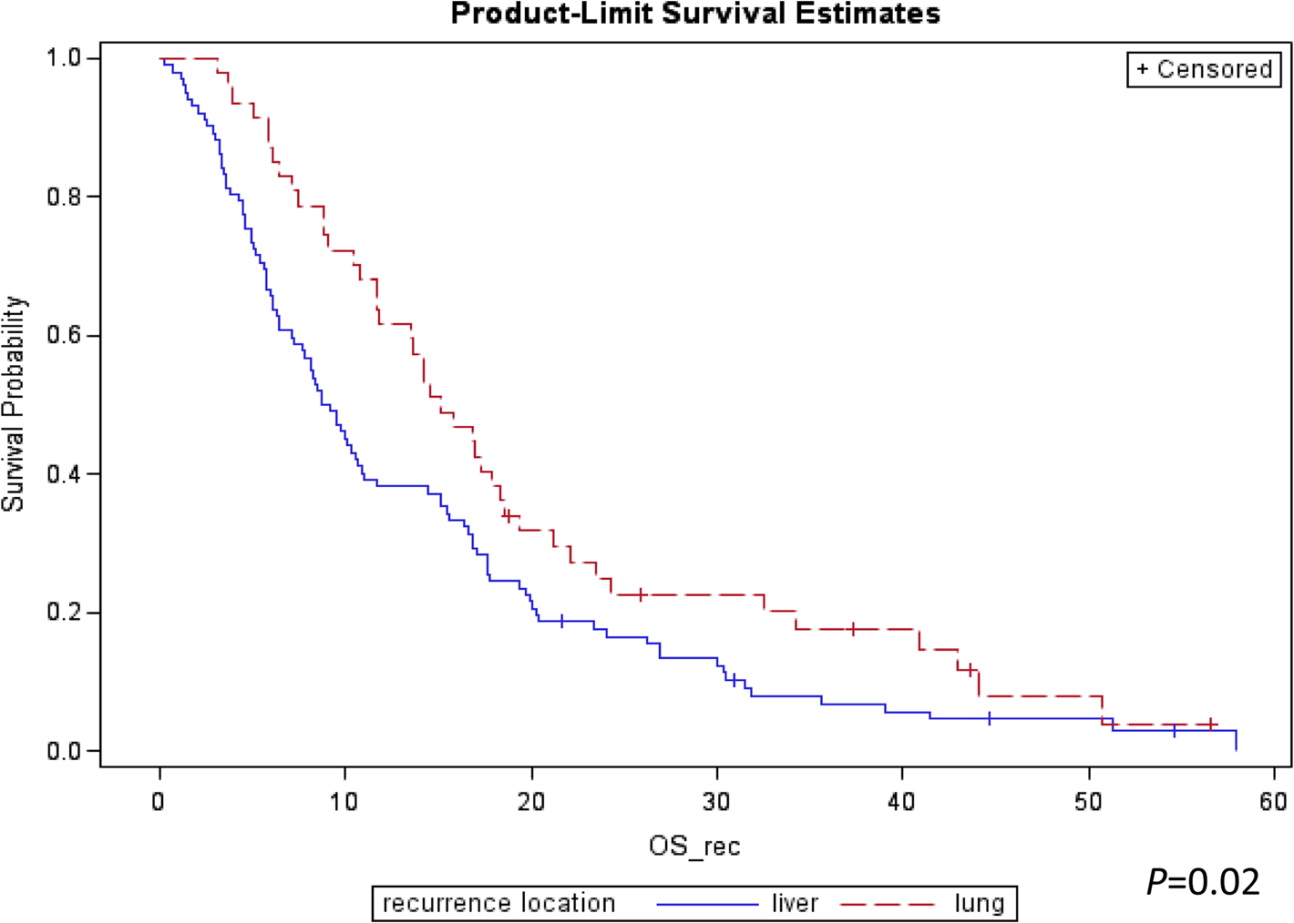
Kaplan Meier Curve for Survival for Liver versus Lung Recurrence

We observed *N* = 140 deaths by January 2016 with a median follow up 37.3 months among survivors. The median TRD was 10.7 months in the overall cohort (95%CI: 8.9–14.6). The median TRD in PDAC patients with lung recurrence as the first site of metastasis compared to patients who recurred initially with liver metastasis was 15 [95%CI 11–18] versus 9 [95%CI: 7–11] months respectively (*P* = 0.02; Fig. 2). One-year and 2 year-survival rates from time of relapse were 38.2% [95% CI: 28–47%] and 17.5% [95% CI: 11–26%] in the liver metastasis cohort and 61.7% [95% CI: 46–74%] and 24.9% [95% CI: 13–38%] in the lung metastasis cohort. In a comparative analysis, underweight status (BMI < 18) was noted to be more common in the lung metastasis group whereas overweight (BMI > 25) and obesity (BMI > 30) were more prevalent in the liver metastasis group at the time of initial diagnosis (Table 1). For the liver metastasis cohort, there was significantly more moderate-poor or poorly differentiated PDAC’s compared to patients with lung metastasis (*N* = 40 (40%) vs *N* = 10 (21%) respectively, *P* = 0.047). We observed a trend for more frequent tumor involvement in lymph nodes at the time of surgery in patients who had lung versus liver metastasis as the first site of recurrence (87% vs 75%, *P* = 0.089) (Table 2). Patients with initial liver recurrence were more likely to have a positive margin resection compared to lung recurrence cohort, however, this difference was not statistically significant (87% vs 77%, *P* = 0.148). We also observed a slightly increased rate of perineural invasion in the lung metastasis cohort compared to the liver metastasis cohort, although again not statistically significant (96% vs 88% *P* = 0.226). Sixty-eight percent of the lung metastasis cohort had a history of either social or daily-base alcohol use in contrast to 45% in the liver metastasis cohort (*P* = 0.011). There was no significant association between the recurrence pattern of PDAC and vascular invasion, site of primary tumor location nor a history of cigarette smoking (Table 2).

## Discussion

In this study, we observed a favorable clinical course with a prolonged time from recurrence to death (TRD) in PDAC patients who underwent curative surgical resection and had subsequent lung metastasis as the first site of recurrence compared to patients who developed recurrent disease in their liver as the first site of metastasis (*P* = 0.02). The difference in clinical course was also supported by the 1 and 2-year survival outcomes. We also found that poorly differentiated PDAC was more likely to recur in the liver rather than lung (*P* = 0.047). In addition, our data suggests that there may be an association between the use of alcohol and the first site of recurrence in PDAC, with lung metastasis compared to liver metastasis as first site of recurrence (*P* = 0.011).

These findings are indicative of a distinct clinical course of PDAC based on the site of recurrence in metastatic disease. The observations reported herein may potentially impact the risk stratification and standard treatment approach of metastatic PDAC. For example, patients who have oligometastatic disease in the lung may be subject to less intensive management, e.g., single or doublet agents therapy in contrast to triplet therapy) whereas patients with liver recurrence may benefit from an intensified treatment approach. Moreover, isolated lung metastases in PDAC can be considered for pulmonary resection with curative intent in a small number of selected patients [10]. The diverse behavior of PDAC based on recurrence pattern may also need to be taken into consideration for stratification in clinical trials including patients with prior surgical resection. Our findings should be further investigated in prospective analyses to confirm the observations.

In our study, we found that moderate to poor and poorly differentiated tumors were associated with an increased incidence of liver metastasis (Table 2). It is unclear, what molecular drivers in poorly differentiated tumors lead to liver metastasis. The relatively better outcomes we reported in regular alcohol users could be explained by the occurrence of different driver (founder) mutations in this patient population compared to the general population. Further genomic analyses based on epidemiological risk factors may shed light on different carcinogenesis pathways with different driver genes in PDAC. We further observed that there may be an increased incidence of liver metastasis in patients with a higher BMI. This association could be related to the direct metabolic effects of increased body weight on liver parenchyma such as fatty liver [11] and increased inflammation such as in non-alcoholic steatohepatitis (NASH) [12]. Given that chronic inflammation provides a safe haven for cancer cell proliferation [13], the liver may be a preferred site by cancer clones in patients with increased BMI. Further, prospective study is warranted to confirm these observations. Lastly, we observed a trend to the increased presence of lung metastasis in patients with lymph node positive disease putatively may be explained by lymphatic drainage of pancreatic lymph nodes into the thoracic duct which drains into the subclavian vein and eventually pulmonary arterial system. This circulatory system may provide a direct pathway for lung metastasis for cancer cells entering into the lymphatic system compared to cancer cells metastasizing via hematogenous spread. Further studies are needed to better understand the underpinnings of these findings.

**Fig. 3:**
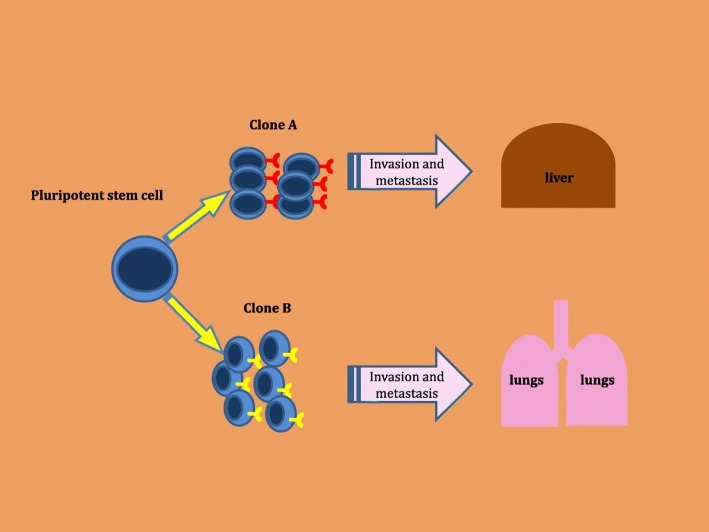
Evolution of Metastasis. Stem cells give rise to new clones with differing features, including distinct chemokine receptors leading to diverse patterns of disease behavior

The observations reported herein may be supported by multiple molecular mechanisms which are involved in the metastatic process. Metastasis is a very sophisticated process influenced by myriad factors such as the molecular behavior of cancer cells, the tumor microenvironment, the immune response to circulating cancer cells and the response of homing organs to the metastatic clones. Metastasis evolves via reprogramming of cancer cells, migration and invasion into stroma, evasion of the immune system, entry into the systemic circulation, colonization in a new tumor environment and homing [14]. This multistep progress may require an extended time period. A study by Yachida, et al., reported that the evolution of a PDAC cell with metastatic features requires considerable genetic modification and requires approximately 5 years from the birth of the parental cancer cell (non-metastatic founder cancer cell) [15]. In fact, throughout this complex process many cancer clones in the primary tumor site undergo either apoptosis or necrosis without achieving metastatic potential to disseminate and propagate in distant organs [16, 17]. Growing evidence suggests that metastatic disease in different distant organs may display varied molecular signatures and diverse clinical behavior. For example, one study explored genetic alterations in *N* = 13 PDAC patients with metastatic disease and found progressive genetic rearrangements in DNA leading to generation of distinct clones with unique mutational signatures among different metastases suggesting that tumor heterogeneity continuously evolves even after evolution of the metastatic process [18] (Fig. 3). Current evidence suggests that tissue hypoxia could be one of the important drivers of these genetic rearrangements that yields to multi-clonal expansion and activation of the metastasis process [19, 20]. Growth of cancer cells in the tumor environment is disproportionate to the nutritional and oxygen supply leading to changes in the metabolism of cancer cells including excessive use of glycolysis called the ‘Warburg effect’ [21]. These imbalances and dynamic changes within the microenvironment yield modifications in the gene expression profile of cancer cells [22]. It is likely that cancer cells continue to conform and modulate their gene expressions in distant metastatic organs including the liver and lungs [18]. The differences in clinical course of disease in liver versus lung metastasis observed in our study could be directly related to the degree of hypoxia in the distant organ along with other characteristics of the metastatic niche. Whether special characteristics of organ-specific homing [23] have an impact on progression of disease in the metastatic setting needs to be further investigated.

## Conclusion

In summary, we observed a trend towards improved outcome in PDAC patients with lung metastasis as the first site of recurrence compared to patients with liver metastasis as the first site of recurrence following potentially curative resection. These observations are partly explained by the heterogeneity of PDAC, the distinct features of the homing microenvironment, and the impact of clonal selection by the chemokine receptor network and other processes. Our results also suggest that the epidemiologic context may be a determinant for disease behavior and recurrence pattern. Our study is limited by several factors including, the potential impact of adjuvant and subsequent treatment for metastasis, abstraction of data from a single institution database, the retrospective nature of cohort and the relatively small size of the patient cohort. Further prospective studies are warranted to interrogate our findings and to further characterize the biologic behavior of PDAC.

## Abbreviations

BMI: Body-mass index
FOLFRINOX: 5-fluorouracil, oxaliplatin, irinotecan, leucovorin
IRB: Institutional Review and Privacy Board
NASH: Non-alcoholic steatohepatitis
PDAC: Pancreatic ductal adenocarcinoma
SD: Standard deviation
TRD: Time from relapse to death
